# Potassium *N*-bromo-2-methyl­benzene­sulfonamidate sesquihydrate

**DOI:** 10.1107/S1600536811025153

**Published:** 2011-07-02

**Authors:** B. Thimme Gowda, Sabine Foro, K. Shakuntala

**Affiliations:** a Department of Chemistry, Mangalore University, Mangalagangotri 574 199, Mangalore, India; b Institute of Materials Science, Darmstadt University of Technology, Petersenstrasse 23, D-64287 Darmstadt, Germany

## Abstract

In the structure of the title compound, K^+^·C_7_H_7_BrNO_2_S^−^·1.5H_2_O, the K^+^ ion is hepta­coordinated by three O atoms from water mol­ecules and by four sulfonyl O atoms of *N*-bromo-2-methyl­benzene­sulfonamide anions. The S—N distance of 1.577 (5) Å is consistent with an S=N double bond. The crystal structure comprises sheets in the *ac* plane which are further stabilized by O—H⋯Br and O—H⋯N hydrogen bonds.

## Related literature

For the preparation of *N*-bromo­aryl­sulfonamides, see: Usha & Gowda (2006 ▶). For our studies of the effect of substituents on the structures of *N*-haloaryl­sulfonamides, see: Gowda & Kumar (2003 ▶); Gowda *et al.* (2009 ▶, 2011 ▶); Usha & Gowda (2006 ▶). For related structures, see: George *et al.* (2000 ▶); Olmstead & Power (1986 ▶).
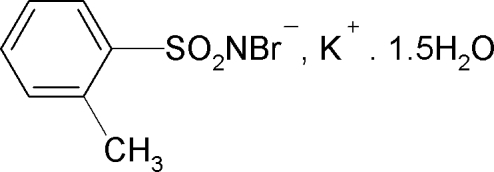


## Experimental

### 

#### Crystal data

K^+^·C_7_H_7_BrNO_2_S^−^·1.5H_2_O
*M* *_r_* = 315.23
Orthorhombic, 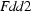

*a* = 12.271 (2) Å
*b* = 55.017 (6) Å
*c* = 6.904 (1) Å
*V* = 4661.0 (11) Å^3^
*Z* = 16
Mo *K*α radiation
μ = 4.05 mm^−1^
*T* = 293 K
0.42 × 0.42 × 0.30 mm

#### Data collection

Oxford Diffraction Xcalibur diffractometer with Sapphire CCD detector
Absorption correction: multi-scan (*CrysAlis RED*; Oxford Diffraction, 2009 ▶) *T*_min_ = 0.281, *T*_max_ = 0.376
7816 measured reflections
2358 independent reflections
2140 reflections with *I* > 2σ(*I*)
*R*_int_ = 0.047

#### Refinement

*R*[*F* ^2^ > 2σ(*F* ^2^)] = 0.048
*wR*(*F* ^2^) = 0.112
*S* = 1.13
2358 reflections
142 parameters
4 restraints
H atoms treated by a mixture of independent and constrained refinement
Δρ_max_ = 0.68 e Å^−3^
Δρ_min_ = −0.57 e Å^−3^
Absolute structure: Flack (1983 ▶), 1060 Friedel pairs
Flack parameter: −0.002 (14)

### 

Data collection: *CrysAlis CCD* (Oxford Diffraction, 2009 ▶); cell refinement: *CrysAlis RED* (Oxford Diffraction, 2009 ▶); data reduction: *CrysAlis RED*; program(s) used to solve structure: *SHELXS97* (Sheldrick, 2008 ▶); program(s) used to refine structure: *SHELXL97* (Sheldrick, 2008 ▶); molecular graphics: *PLATON* (Spek, 2009 ▶); software used to prepare material for publication: *SHELXL97*.

## Supplementary Material

Crystal structure: contains datablock(s) I, global. DOI: 10.1107/S1600536811025153/ci5191sup1.cif

Structure factors: contains datablock(s) I. DOI: 10.1107/S1600536811025153/ci5191Isup2.hkl

Additional supplementary materials:  crystallographic information; 3D view; checkCIF report

## Figures and Tables

**Table 1:** Hydrogen-bond geometry (Å, °)

*D*—H⋯*A*	*D*—H	H⋯*A*	*D*⋯*A*	*D*—H⋯*A*
O3—H31⋯Br1^i^	0.81 (2)	2.79 (2)	3.600 (5)	173 (7)
O3—H32⋯N1^ii^	0.81 (2)	2.19 (4)	2.933 (7)	154 (7)
O4—H41⋯N1^iii^	0.80 (2)	2.28 (5)	2.993 (6)	149 (8)

Symmetry codes: (i) 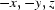; (ii) 
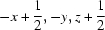
; (iii) 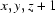.

**Fig. 1.**
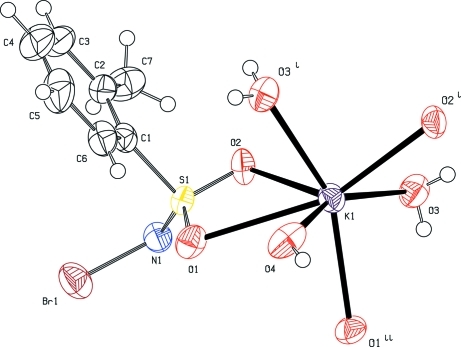
Molecular structure of the title compound, showing the atom labelling scheme for the asymmetric unit and extended to show the coordination geometry for the K+ cation. Displacement ellipsoids are drawn at the 50% probability level and H atoms are represented as small spheres of arbitrary radii. Symmetry codes: (i) 1/2-x, -y, 1/2+z and (ii) -x,-y,z.

**Fig. 2.**
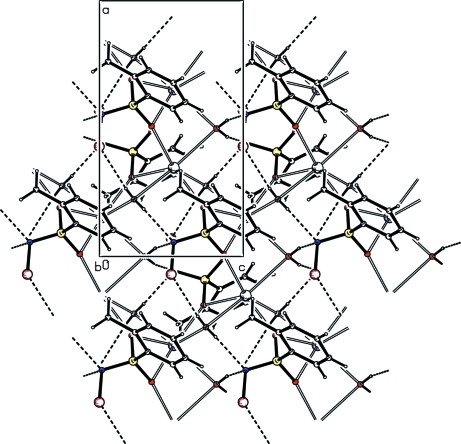
Molecular packing of the title compound with hydrogen bonds shown as dashed lines.

## supplementary crystallographic information

### Comment

To explore the substituent effects and the effect of replacing sodium ion by potassium ion on the solid state structures of *N*-halo-arylsulfonamidates (Gowda & Kumar, 2003; Gowda *et al.*, 2009, 2011; Usha & Gowda, 2006), in the present work, the structure of potassium *N*-bromo-2-methyl-benzenesulfonamidate sesquihydrate (I) has been determined (Fig. 1). The structure of (I) resembles those of potassium *N*-bromo-2-chloro-benzenesulfonamidate sesquihydrate (II) (Gowda *et al.*, 2011), sodium *N*-chloro-2-methyl- benzenesulfonamidate sesquihydrate (III) (Gowda *et al.*, 2009), and other sodium *N*-chloro-arylsulfonamidates (George *et al.*, 2000; Olmstead & Power, 1986).

In the title compound, K^+^ ion is hepta coordinated by three O atoms from water molecules and by four sulfonyl O atoms of *N*-bromo-2-methyl- benzenesulfonamide anions. The replacement of Na^+^ ion by K^+^ ion changes co-ordination from hexa to hepta in the metal co-ordination (Gowda *et al.*, 2009) and other parameters.

The S—N distance of 1.577 (5) Å is consistent with an S—N double bond and is in agreement with the observed values of 1.582 (4)Å in (II) and 1.590 (2) Å in (III).

The packing consists of two-dimensional polymeric layers running parallel to the *ac* plane (Fig. 2). The molecular packing is stabilized by O3—H31···Br1, O3—H32···N1 and O4—H41···N1 hydrogen bonds (Table 1).

### Experimental

The title compound was prepared according to the literature method (Usha & Gowda, 2006). The purity of the compound was checked by determining its melting point (176 °). It was characterized by recording its infrared and NMR spectra. Yellow prisms of the title compound used in X-ray diffraction studies were obtained from its aqueous solution at room temperature.

### Refinement

The O bound H atoms were located in a difference map and later restrained to O–H = 0.82 (2) Å. The other H atoms were positioned with idealized geometry using a riding model with aromatic C–H = 0.93 Å and methyl C–H = 0.96 Å. All H atoms were refined with isotropic displacement parameters (set to 1.2 times of the *U*_eq_ of the parent atom).

### Crystal data

**Table:**

K^+^·C_7_H_7_BrNO_2_S^−^·1.5H_2_O	*F*(000) = 2512
*M**_r_* = 315.23	*D*_x_ = 1.797 Mg m^−^^3^
Orthorhombic, *F**d**d*2	Mo *K*α radiation, λ = 0.71073 Å
Hall symbol: F 2 -2d	Cell parameters from 5298 reflections
*a* = 12.271 (2) Å	θ = 2.8–27.9°
*b* = 55.017 (6) Å	µ = 4.05 mm^−^^1^
*c* = 6.904 (1) Å	*T* = 293 K
*V* = 4661.0 (11) Å^3^	Prism, yellow
*Z* = 16	0.42 × 0.42 × 0.30 mm

### Data collection

**Table:**

Oxford Diffraction Xcalibur diffractometer with Sapphire CCD detector	2358 independent reflections
Radiation source: fine-focus sealed tube	2140 reflections with *I* > 2σ(*I*)
graphite	*R*_int_ = 0.047
Rotation method data acquisition using ω scans	θ_max_ = 26.4°, θ_min_ = 3.0°
Absorption correction: multi-scan (*CrysAlis RED*; Oxford Diffraction, 2009)	*h* = −15→15
*T*_min_ = 0.281, *T*_max_ = 0.376	*k* = −61→68
7816 measured reflections	*l* = −8→8

### Refinement

**Table:**

Refinement on *F*^2^	Secondary atom site location: difference Fourier map
Least-squares matrix: full	Hydrogen site location: inferred from neighbouring sites
*R*[*F*^2^ > 2σ(*F*^2^)] = 0.048	H atoms treated by a mixture of independent and constrained refinement
*wR*(*F*^2^) = 0.112	*w* = 1/[σ^2^(*F*_o_^2^) + (0.0467*P*)^2^ + 17.9942*P*] where *P* = (*F*_o_^2^ + 2*F*_c_^2^)/3
*S* = 1.13	(Δ/σ)_max_ = 0.002
2358 reflections	Δρ_max_ = 0.68 e Å^−^^3^
142 parameters	Δρ_min_ = −0.57 e Å^−^^3^
4 restraints	Absolute structure: Flack (1983), 1060 Friedel pairs
Primary atom site location: structure-invariant direct methods	Flack parameter: −0.002 (14)

### Special details

**Table:**

Geometry. All e.s.d.'s (except the e.s.d. in the dihedral angle between two l.s. planes) are estimated using the full covariance matrix. The cell e.s.d.'s are taken into account individually in the estimation of e.s.d.'s in distances, angles and torsion angles; correlations between e.s.d.'s in cell parameters are only used when they are defined by crystal symmetry. An approximate (isotropic) treatment of cell e.s.d.'s is used for estimating e.s.d.'s involving l.s. planes.
Refinement. Refinement of *F*^2^ against ALL reflections. The weighted *R*-factor *wR* and goodness of fit *S* are based on *F*^2^, conventional *R*-factors *R* are based on *F*, with *F* set to zero for negative *F*^2^. The threshold expression of *F*^2^ > σ(*F*^2^) is used only for calculating *R*-factors(gt) *etc*. and is not relevant to the choice of reflections for refinement. *R*-factors based on *F*^2^ are statistically about twice as large as those based on *F*, and *R*- factors based on ALL data will be even larger.

### Fractional atomic coordinates and isotropic or equivalent isotropic displacement parameters (Å^2^)

**Table:**

	*x*	*y*	*z*	*U*_iso_*/*U*_eq_	
Br1	−0.06968 (5)	0.066623 (13)	0.49885 (10)	0.0532 (2)	
K1	0.15329 (9)	−0.00653 (2)	1.01716 (18)	0.0354 (3)	
S1	0.09151 (10)	0.04335 (2)	0.73436 (19)	0.0285 (3)	
O1	0.0096 (3)	0.03331 (7)	0.8626 (6)	0.0411 (10)	
O2	0.1906 (3)	0.02885 (7)	0.7241 (7)	0.0395 (9)	
O3	0.2761 (4)	−0.03137 (8)	0.7404 (8)	0.0441 (10)	
H31	0.234 (5)	−0.0403 (11)	0.683 (9)	0.053*	
H32	0.314 (5)	−0.0400 (11)	0.807 (9)	0.053*	
O4	0.0000	0.0000	1.3142 (9)	0.0507 (17)	
H41	0.031 (6)	0.0087 (12)	1.388 (9)	0.061*	
N1	0.0555 (3)	0.04649 (9)	0.5166 (7)	0.0361 (11)	
C1	0.1275 (5)	0.07197 (11)	0.8363 (8)	0.0334 (12)	
C2	0.2037 (4)	0.08730 (11)	0.7475 (11)	0.0401 (13)	
C3	0.2281 (6)	0.10908 (13)	0.8424 (12)	0.0561 (19)	
H3	0.2786	0.1196	0.7877	0.067*	
C4	0.1789 (6)	0.11525 (14)	1.0151 (14)	0.070 (2)	
H4	0.1968	0.1298	1.0760	0.084*	
C5	0.1040 (7)	0.10007 (15)	1.0968 (12)	0.064 (2)	
H5	0.0700	0.1045	1.2120	0.077*	
C6	0.0784 (5)	0.07831 (11)	1.0103 (10)	0.0427 (14)	
H6	0.0284	0.0679	1.0680	0.051*	
C7	0.2617 (6)	0.08176 (15)	0.5606 (11)	0.0579 (19)	
H7A	0.2225	0.0889	0.4546	0.070*	
H7B	0.2656	0.0645	0.5428	0.070*	
H7C	0.3341	0.0884	0.5651	0.070*	

### Atomic displacement parameters (Å^2^)

**Table:**

	*U*^11^	*U*^22^	*U*^33^	*U*^12^	*U*^13^	*U*^23^
Br1	0.0458 (3)	0.0596 (4)	0.0542 (4)	0.0077 (3)	−0.0059 (3)	0.0155 (4)
K1	0.0369 (6)	0.0350 (7)	0.0342 (6)	0.0050 (5)	−0.0057 (6)	−0.0007 (5)
S1	0.0349 (6)	0.0215 (6)	0.0292 (6)	0.0011 (5)	0.0023 (6)	0.0019 (5)
O1	0.047 (2)	0.035 (3)	0.041 (2)	−0.0050 (19)	0.0112 (18)	0.0067 (19)
O2	0.044 (2)	0.032 (2)	0.043 (2)	0.0123 (18)	0.007 (2)	0.008 (2)
O3	0.054 (2)	0.036 (2)	0.042 (2)	0.0000 (19)	−0.003 (2)	−0.002 (2)
O4	0.078 (5)	0.042 (4)	0.032 (3)	−0.018 (3)	0.000	0.000
N1	0.041 (2)	0.037 (3)	0.031 (2)	0.006 (2)	−0.006 (2)	−0.008 (2)
C1	0.038 (3)	0.031 (3)	0.031 (3)	0.006 (2)	−0.009 (2)	0.002 (2)
C2	0.040 (3)	0.032 (3)	0.048 (3)	−0.001 (2)	−0.009 (3)	0.005 (3)
C3	0.063 (4)	0.035 (4)	0.070 (5)	−0.010 (3)	−0.023 (4)	0.003 (4)
C4	0.084 (5)	0.044 (5)	0.081 (6)	0.001 (4)	−0.030 (5)	−0.024 (4)
C5	0.089 (6)	0.051 (5)	0.053 (4)	0.021 (4)	−0.011 (4)	−0.016 (4)
C6	0.054 (3)	0.042 (3)	0.032 (3)	0.012 (3)	−0.007 (3)	−0.011 (3)
C7	0.065 (4)	0.057 (5)	0.051 (4)	−0.020 (4)	−0.001 (3)	0.008 (3)

### Geometric parameters (Å, °)

**Table:**

Br1—N1	1.897 (4)	O3—H31	0.81 (2)
K1—O2^i^	2.687 (4)	O3—H32	0.81 (2)
K1—O1^ii^	2.703 (4)	O4—K1^ii^	2.806 (5)
K1—O3^i^	2.734 (5)	O4—H41	0.80 (2)
K1—O3	2.791 (5)	C1—C6	1.388 (9)
K1—O4	2.806 (5)	C1—C2	1.401 (8)
K1—O2	2.845 (4)	C2—C3	1.398 (9)
K1—O1	3.009 (4)	C2—C7	1.505 (10)
K1—S1	3.4522 (17)	C3—C4	1.379 (12)
K1—H32	3.06 (7)	C3—H3	0.93
K1—H41	3.08 (7)	C4—C5	1.364 (12)
S1—O1	1.448 (4)	C4—H4	0.93
S1—O2	1.456 (4)	C5—C6	1.375 (10)
S1—N1	1.577 (5)	C5—H5	0.93
S1—C1	1.780 (6)	C6—H6	0.93
O1—K1^ii^	2.703 (4)	C7—H7A	0.96
O2—K1^iii^	2.687 (4)	C7—H7B	0.96
O3—K1^iii^	2.734 (5)	C7—H7C	0.96
			
O2^i^—K1—O1^ii^	119.23 (13)	O1—S1—C1	105.5 (3)
O2^i^—K1—O3^i^	79.79 (13)	O2—S1—C1	107.3 (3)
O1^ii^—K1—O3^i^	150.77 (14)	N1—S1—C1	110.4 (3)
O2^i^—K1—O3	75.84 (15)	O1—S1—K1	60.24 (17)
O1^ii^—K1—O3	82.08 (13)	O2—S1—K1	53.75 (17)
O3^i^—K1—O3	126.03 (8)	N1—S1—K1	133.49 (18)
O2^i^—K1—O4	98.51 (14)	C1—S1—K1	115.16 (18)
O1^ii^—K1—O4	82.10 (12)	S1—O1—K1^ii^	164.2 (3)
O3^i^—K1—O4	72.71 (11)	S1—O1—K1	95.1 (2)
O3—K1—O4	157.69 (11)	K1^ii^—O1—K1	84.04 (11)
O2^i^—K1—O2	125.18 (8)	S1—O2—K1^iii^	150.7 (3)
O1^ii^—K1—O2	102.19 (14)	S1—O2—K1	101.9 (2)
O3^i^—K1—O2	80.10 (15)	K1^iii^—O2—K1	100.39 (12)
O3—K1—O2	76.18 (13)	K1^iii^—O3—K1	100.59 (15)
O4—K1—O2	122.68 (10)	K1^iii^—O3—H31	113 (5)
O2^i^—K1—O1	159.94 (13)	K1—O3—H31	107 (5)
O1^ii^—K1—O1	79.85 (15)	K1^iii^—O3—H32	126 (5)
O3^i^—K1—O1	80.21 (13)	K1—O3—H32	102 (5)
O3—K1—O1	115.48 (14)	H31—O3—H32	107 (7)
O4—K1—O1	76.89 (11)	K1^ii^—O4—K1	86.09 (18)
O2—K1—O1	48.96 (11)	K1^ii^—O4—H41	135 (6)
O2^i^—K1—S1	145.14 (11)	K1—O4—H41	103 (6)
O1^ii^—K1—S1	92.72 (10)	S1—N1—Br1	110.7 (3)
O3^i^—K1—S1	77.42 (11)	C6—C1—C2	121.1 (6)
O3—K1—S1	96.92 (11)	C6—C1—S1	117.2 (5)
O4—K1—S1	99.45 (7)	C2—C1—S1	121.7 (5)
O2—K1—S1	24.37 (8)	C3—C2—C1	117.0 (7)
O1—K1—S1	24.70 (8)	C3—C2—C7	118.3 (6)
O2^i^—K1—H32	61.2 (8)	C1—C2—C7	124.7 (6)
O1^ii^—K1—H32	87.8 (13)	C4—C3—C2	121.5 (7)
O3^i^—K1—H32	121.4 (13)	C4—C3—H3	119.2
O3—K1—H32	15.0 (7)	C2—C3—H3	119.2
O4—K1—H32	148.7 (11)	C5—C4—C3	120.1 (7)
O2—K1—H32	88.3 (10)	C5—C4—H4	119.9
O1—K1—H32	130.3 (7)	C3—C4—H4	119.9
S1—K1—H32	110.6 (9)	C4—C5—C6	120.5 (8)
O2^i^—K1—H41	91.7 (14)	C4—C5—H5	119.7
O1^ii^—K1—H41	96.7 (8)	C6—C5—H5	119.7
O3^i^—K1—H41	58.6 (8)	C5—C6—C1	119.7 (7)
O3—K1—H41	164.7 (15)	C5—C6—H6	120.1
O4—K1—H41	14.6 (7)	C1—C6—H6	120.1
O2—K1—H41	118.9 (14)	C2—C7—H7A	109.5
O1—K1—H41	79.1 (15)	C2—C7—H7B	109.5
S1—K1—H41	98.4 (15)	H7A—C7—H7B	109.5
H32—K1—H41	150.4 (17)	C2—C7—H7C	109.5
O1—S1—O2	113.6 (3)	H7A—C7—H7C	109.5
O1—S1—N1	115.5 (3)	H7B—C7—H7C	109.5
O2—S1—N1	104.4 (3)		
			
O2^i^—K1—S1—O1	−144.3 (3)	C1—S1—O2—K1	108.9 (2)
O1^ii^—K1—S1—O1	58.28 (18)	O2^i^—K1—O2—S1	−151.27 (18)
O3^i^—K1—S1—O1	−93.9 (2)	O1^ii^—K1—O2—S1	69.0 (3)
O3—K1—S1—O1	140.6 (2)	O3^i^—K1—O2—S1	−81.3 (3)
O4—K1—S1—O1	−24.2 (2)	O3—K1—O2—S1	147.5 (3)
O2—K1—S1—O1	172.3 (3)	O4—K1—O2—S1	−19.4 (3)
O2^i^—K1—S1—O2	43.4 (2)	O1—K1—O2—S1	4.25 (18)
O1^ii^—K1—S1—O2	−114.0 (3)	O2^i^—K1—O2—K1^iii^	48.0 (2)
O3^i^—K1—S1—O2	93.8 (3)	O1^ii^—K1—O2—K1^iii^	−91.81 (15)
O3—K1—S1—O2	−31.7 (3)	O3^i^—K1—O2—K1^iii^	117.89 (15)
O4—K1—S1—O2	163.5 (3)	O3—K1—O2—K1^iii^	−13.25 (13)
O1—K1—S1—O2	−172.3 (3)	O4—K1—O2—K1^iii^	179.83 (12)
O2^i^—K1—S1—N1	117.7 (3)	O1—K1—O2—K1^iii^	−156.5 (2)
O1^ii^—K1—S1—N1	−39.8 (3)	S1—K1—O2—K1^iii^	−160.8 (3)
O3^i^—K1—S1—N1	168.0 (3)	O2^i^—K1—O3—K1^iii^	−119.35 (16)
O3—K1—S1—N1	42.6 (3)	O1^ii^—K1—O3—K1^iii^	117.72 (16)
O4—K1—S1—N1	−122.2 (3)	O3^i^—K1—O3—K1^iii^	−53.5 (3)
O2—K1—S1—N1	74.2 (3)	O4—K1—O3—K1^iii^	162.9 (3)
O1—K1—S1—N1	−98.1 (3)	O2—K1—O3—K1^iii^	13.02 (13)
O2^i^—K1—S1—C1	−50.1 (3)	O1—K1—O3—K1^iii^	42.99 (18)
O1^ii^—K1—S1—C1	152.4 (2)	S1—K1—O3—K1^iii^	25.92 (13)
O3^i^—K1—S1—C1	0.2 (2)	O2^i^—K1—O4—K1^ii^	−161.43 (10)
O3—K1—S1—C1	−125.3 (2)	O1^ii^—K1—O4—K1^ii^	−42.89 (9)
O4—K1—S1—C1	70.0 (2)	O3^i^—K1—O4—K1^ii^	122.12 (11)
O2—K1—S1—C1	−93.6 (3)	O3—K1—O4—K1^ii^	−88.1 (4)
O1—K1—S1—C1	94.1 (3)	O2—K1—O4—K1^ii^	56.56 (13)
O2—S1—O1—K1^ii^	−79.2 (10)	O1—K1—O4—K1^ii^	38.46 (9)
N1—S1—O1—K1^ii^	41.3 (10)	S1—K1—O4—K1^ii^	48.57 (3)
C1—S1—O1—K1^ii^	163.5 (9)	O1—S1—N1—Br1	57.7 (3)
K1—S1—O1—K1^ii^	−86.0 (9)	O2—S1—N1—Br1	−176.9 (2)
O2—S1—O1—K1	6.8 (3)	C1—S1—N1—Br1	−61.9 (3)
N1—S1—O1—K1	127.3 (2)	K1—S1—N1—Br1	129.90 (18)
C1—S1—O1—K1	−110.5 (2)	O1—S1—C1—C6	2.8 (5)
O2^i^—K1—O1—S1	76.8 (5)	O2—S1—C1—C6	−118.6 (5)
O1^ii^—K1—O1—S1	−120.32 (14)	N1—S1—C1—C6	128.3 (4)
O3^i^—K1—O1—S1	81.2 (2)	K1—S1—C1—C6	−61.2 (5)
O3—K1—O1—S1	−44.2 (2)	O1—S1—C1—C2	−178.5 (5)
O4—K1—O1—S1	155.5 (2)	O2—S1—C1—C2	60.1 (5)
O2—K1—O1—S1	−4.20 (18)	N1—S1—C1—C2	−53.1 (5)
O2^i^—K1—O1—K1^ii^	−119.1 (4)	K1—S1—C1—C2	117.5 (4)
O1^ii^—K1—O1—K1^ii^	43.81 (17)	C6—C1—C2—C3	0.3 (8)
O3^i^—K1—O1—K1^ii^	−114.72 (14)	S1—C1—C2—C3	−178.4 (5)
O3—K1—O1—K1^ii^	119.90 (14)	C6—C1—C2—C7	179.3 (6)
O4—K1—O1—K1^ii^	−40.36 (9)	S1—C1—C2—C7	0.6 (8)
O2—K1—O1—K1^ii^	159.9 (2)	C1—C2—C3—C4	−0.3 (10)
S1—K1—O1—K1^ii^	164.1 (3)	C7—C2—C3—C4	−179.4 (7)
O1—S1—O2—K1^iii^	131.3 (5)	C2—C3—C4—C5	−0.5 (11)
N1—S1—O2—K1^iii^	4.7 (6)	C3—C4—C5—C6	1.3 (12)
C1—S1—O2—K1^iii^	−112.5 (5)	C4—C5—C6—C1	−1.3 (11)
K1—S1—O2—K1^iii^	138.6 (6)	C2—C1—C6—C5	0.6 (9)
O1—S1—O2—K1	−7.3 (3)	S1—C1—C6—C5	179.3 (5)
N1—S1—O2—K1	−133.9 (2)		

Symmetry codes: (i) −*x*+1/2, −*y*, *z*+1/2; (ii) −*x*, −*y*, *z*; (iii) −*x*+1/2, −*y*, *z*−1/2.

### Hydrogen-bond geometry (Å, °)

**Table:**

*D*—H···*A*	*D*—H	H···*A*	*D*···*A*	*D*—H···*A*
O3—H31···Br1^ii^	0.81 (2)	2.79 (2)	3.600 (5)	173 (7)
O3—H32···N1^i^	0.81 (2)	2.19 (4)	2.933 (7)	154 (7)
O4—H41···N1^iv^	0.80 (2)	2.28 (5)	2.993 (6)	149 (8)

Symmetry codes: (ii) −*x*, −*y*, *z*; (i) −*x*+1/2, −*y*, *z*+1/2; (iv) *x*, *y*, *z*+1.
